# The Economy of Different Types Compared

**Published:** 1912-02-24

**Authors:** H. Hyslop Thomson

**Affiliations:** Medical Superintendent Liverpool Sanatorium, Frodsham, Cheshire.


### SANATORIUM CONSTRUCTION.

### The Economy of Different Types Compared.

By H. HYSLOP THOMSON, M.D., D.P.H., Medical Superintendent Liverpool Sanatorium, Frodsham, Cheshire.

The passing of the National Insurance Bill has focussed public attention on the most efficient and economical means of treating tuberculosis by modern sanatorium methods. In the past mistakes have been made in spending too much money on the structural portion of the sanatorium without any commensurate gain in efficiency, and the fact has frequently been ignored that treatment can efficiently be carried out under simpler and less expensive conditions. In consequence of this mistaken view not a few sanatoriums have been erected which have cost anything from £500 to £1,000 per bed, a fact which has given rise to much hostile criticism as tending to preclude the possibility of the treatment beng extended to any large number of people. In the present article it is proposed to discuss briefly the sanatorium treatment of tuberculosis from the point of view of economy combined with efficiency.

### Site in Relation to Control.

In erecting a sanatorium attention must first of all be directed to the selection of a suitable and convenient site. Many important points require to be considered and carefully weighed before the site is selected. It is almost unnecessary to emphasise the importance of altitude, aspect, protection from cold winds, dryness of subsoil, etc. The site must be viewed not only as to its suitability for purposes of treatment but also as to the effect it may have on the cost of construction. Two important points are water-supply and sewage disposal. If there is not a sufficient supply of gravitation water the cost of the sanatorium will be increased by the necessity for pumping or boring plant. For this reason it is desirable when possible to select a site in close proximity to the water-main supplying a large town or city. It is also necessary that the site should be selected with a view to secure a natural fall from the sanatorium, and the erection of sewage works at some considerable distance from the building.

Another important point is the absence of any considerable population in the immediate vicinity of the sanatorium. It is desirable, of course, that the sanatorium should be within easy reach of railway facilities, but preferably the station should be an isolated one. If graduated walking exercise is carried on outside the grounds it is imperative that the sanatorium should be somewhat isolated. The necessity for this is obvious. It is not desirable that patients while resident in a sanatorium should be able to procure food or drink during their walks. Again, patients may drop their handkerchiefs when walking outside the grounds, and this will give cause for complaint to those resident in the district. These difficulties, however, can be overcome in large measure by strict rules and careful supervision.

### Types of Construction.

The sanatoriums which have already been erected in this country may be divided for practical consideration into three types, viz. the institutional type, the pavilion type, and the separate chalet type, and it is desirable to consider the relative cost and efficiency of these various types.

### The Institutional.

The institutional type is represented by a large expensive building in which are combined administrative quarters and rooms for the treatment of patients. Such institutions usually have from 60 to 100 patients of both sexes resident in rooms containing from one to four beds. The rooms are placed one above the other in two or sometimes three flats.

Indeed the whole principle is similar to that which obtains in a modern hydropathic, and is unsatisfactory for several reasons. In the first place, the cost is such that it precludes the erection of any adequate number of buildings of this kind. With increase in size and number of floors the cost of a sanatorium of this type rises rapidly without any corresponding increase in efficiency. Some buildings of this kind cost £1,000 per bed. Moreover it is not advisable to combine administrative quarters with the accommodation of a large number of patients in various stages of the disease, as the control and management become more difficult and the cross-ventilation secured is less satisfactory. Lastly, the contrast between a large sanatorium of this type and the home to which the working class patient returns is too great to enable the treatment to be of much educational value.

### The Pavilion and Economy.

The pavilion type of sanatorium consists of a central administrative block which also contains accommodation for a certain number of patients and separate pavilions arranged on each side for the different sexes.

This type of building embodies a high degree of efficiency at a relatively low cost, and a modification of it is the type which the writer recommends for the treatment of tuberculous patients from amongst the industrial classes. Its advantages with regard to efficiency and economy will be considered later.

### The Chalet's Disadvantages.

The separate chalet type consists of a central administrative block and a number of separate chalets for one or more patients distributed throughout the grounds. The chief disadvantages of this type of sanatorium are the cost per head in the treatment of any large number of patients and the difficulty in carrying out efficient control and discipline.

*(To be continued.)*

